# An HLA-Transgenic Mouse Model of Type 1 Diabetes That Incorporates the Reduced but Not Abolished Thymic Insulin Expression Seen in Patients

**DOI:** 10.1155/2016/7959060

**Published:** 2015-12-28

**Authors:** Jeffrey Babad, Riyasat Ali, Jennifer Schloss, Teresa P. DiLorenzo

**Affiliations:** ^1^Department of Microbiology and Immunology, Albert Einstein College of Medicine, Bronx, NY 10461, USA; ^2^Department of Medicine, Division of Endocrinology, Albert Einstein College of Medicine, Bronx, NY 10461, USA

## Abstract

Type 1 diabetes (T1D) is an autoimmune disease characterized by T cell-mediated destruction of the pancreatic islet beta cells. Multiple genetic loci contribute to disease susceptibility in humans, with the most responsible locus being the major histocompatibility complex (MHC). Certain MHC alleles are predisposing, including the common HLA-A^∗^02:01. After the MHC, the locus conferring the strongest susceptibility to T1D is the regulatory region of the insulin gene, and alleles associated with reduced thymic insulin expression are predisposing. Mice express two insulin genes, *Ins1* and *Ins2*. While both are expressed in beta cells, only *Ins2* is expressed in the thymus. We have developed an HLA-A^∗^02:01-transgenic NOD-based T1D model that is heterozygous for a functional *Ins2* gene. These mice exhibit reduced thymic insulin expression and accelerated disease in both genders. Immune cell populations are not grossly altered, and the mice exhibit typical signs of islet autoimmunity, including CD8 T cell responses to beta cell peptides also targeted in HLA-A^∗^02:01-positive type 1 diabetes patients. This model should find utility as a tool to uncover the mechanisms underlying the association between reduced thymic insulin expression and T1D in humans and aid in preclinical studies to evaluate insulin-targeted immunotherapies for the disease.

## 1. Introduction

Type 1 diabetes (T1D) is an autoimmune disease characterized by T cell-mediated destruction of the pancreatic islet beta cells. Multiple genetic loci contribute to T1D susceptibility in humans, with the most responsible locus being the major histocompatibility complex (MHC) [1]. The ability of certain class II MHC genes to influence disease risk has long been appreciated [2, 3]. Multiple studies have also revealed an association with certain class I MHC alleles, including the common HLA-A^∗^02:01 [4–13]. These findings are not surprising, given that CD4 and CD8 T cell responses to a variety of beta cell antigens, including insulin, are observed in T1D patients [14].

After the MHC, the locus that confers the strongest susceptibility to T1D in humans is the variable number of tandem repeats (VNTR) regulatory region of the insulin gene [1, 15]. VNTR alleles with the smallest number of repeats, designated as class I, predispose to T1D [16, 17], while the longer class III alleles have a dominant protective effect [15, 18]. Class III VNTR alleles are associated with thymic insulin RNA levels that are increased two- to threefold compared to class I alleles [19], leading to the hypothesis that impaired negative selection of insulin-specific T cells in individuals with class I VNTR alleles explains their predisposition to T1D [19, 20]. While findings from a single human study are consistent with this idea [21], the development of a mouse model for T1D that incorporates the reduced, but not abolished, thymic insulin expression observed in patients would allow for more rigorous future testing of this hypothesis.

The NOD mouse is the primary rodent model used for studying T1D [22]. Unlike humans, mice express two insulin genes,* Ins1* and* Ins2*. While both genes are expressed in beta cells [23],* Ins2* expression predominates in the thymus [24–27], with little [24] to no [25–27] detectable thymic* Ins1* expression. Ins2-deficient (Ins2^KO^) NOD mice develop diabetes at an accelerated rate [28–30], as do HLA-A^∗^02:01-transgenic Ins2^KO^ NOD mice [28], and both Ins2-deficient strains have increased insulin-specific islet-infiltrating CD8 T cells compared to their wild-type (WT) counterparts [28]. While these Ins2^KO^ mouse strains highlight the importance of thymic insulin expression, they do not accurately represent a human patient, where thymic insulin expression is diminished but still present [19, 20]. Here we have developed an HLA-A^∗^02:01-transgenic NOD-based T1D model that is heterozygous (het) for the Ins2^KO^ allele, resulting in thymic insulin expression that is decreased but not eliminated. The mice develop accelerated disease compared to Ins2^WT^ mice, and this is true regardless of gender. Immune cell populations are not grossly altered, and the mice exhibit typical signs of islet autoimmunity, including CD8 T cell responses to beta cell peptides also targeted in HLA-A^∗^02:01-positive T1D patients. This model should find utility as a tool to uncover the mechanisms underlying the association between class I VNTR alleles and T1D in humans. It should also aid in preclinical studies to evaluate insulin-targeted immunotherapies for the disease.

## 2. Materials and Methods

### 2.1. Mice

NOD.*β*_2_m^KO^.HHD mice [31] transgenically express a single-chain chimeric HLA-A^∗^02:01 molecule in which human *β*2-microglobulin (*β*_2_m) is covalently linked to the *α*1 and *α*2 domains of HLA-A^∗^02:01. The *α*3, transmembrane, and cytoplasmic portions of the molecule are derived from H-2D^b^. Mouse class I MHC molecules are not expressed in these mice due to the murine *β*_2_m deficiency. NOD.Ins2^KO^ mice have been described [29]. The two strains were intercrossed to transfer the Ins2^KO^ allele to the NOD.*β*_2_m^KO^.HHD strain. The resulting progeny were bred as appropriate to obtain Ins2^WT^ and Ins2^het^ NOD.*β*_2_m^KO^.HHD mice for our studies. Female *β*_2_m^KO^ mice breed poorly in our hands and so were rarely used for this purpose. Similarly, NOD and NOD.Ins2^KO^ mice were intercrossed and the resulting progeny bred as appropriate to obtain Ins2^WT^ and Ins2^het^ NOD mice. The HHD transgene and the WT and KO *β*_2_m and Ins2 alleles were identified by PCR using the following primer pairs: HHD, 5′-CTTCATCGCAGTGGGCTAC-3′ and 5′-CGGTGAGTCTGTGAGTGGG-3′; *β*_2_M^WT^, 5′-GAAACCCCTCAAATTCAAGTATACTCA-3′ and 5′-GACGGTCTTGGGCTCGGCCATACT-3′; *β*_2_m^KO^, 5′-GAAACCCCTCAAATTCAAGTATACTCA-3′ and 5′-TCGAATTCGCCAATGACAAGACGCT-3′; Ins2^WT^, 5′-GGCAGAGAGGAGGTGCTTTG-3′ and 5′-AGAAAACCACCAGGGTAGTTAGC-3′; Ins2^KO^, 5′-GGCAGAGAGGAGGTGCTTTG-3′ and 5′-ATTGACCGTAATGGGATAGG-3′. All animal experiments were approved by the Institutional Animal Care and Use Committee of Albert Einstein College of Medicine.

### 2.2. Measurement of Thymic* Ins2* RNA

Female Ins2^WT^ and Ins2^het^ NOD.*β*2m^KO^.HHD mice (four each) were sacrificed and thymus was harvested. Total thymic RNA was isolated using the RNeasy Midi Kit (Qiagen, Valencia, CA) and treated with DNase I (Qiagen) to eliminate DNA contamination. 1.5–2.3 *μ*g of RNA was reverse-transcribed to cDNA using random hexadeoxynucleotides and oligo dT primers (Invitrogen). Equal amounts of cDNA were mixed with SYBR Green PCR Master Mix (Qiagen) and each Ins2 primer (5′-CTTCTTCTACACACCCATGTCC-3′ and 5′-TCTACAATGCCACGCTTCTG-3′) or primers for the U6 normalization control (5′-CTCGCTTCGGCAGCACATATACTA-3′ and 5′-ACGAATTTGCGTGTCATCCTTGCG-3′) and brought to a final volume of 25 *μ*L. Real-time quantitative RT-PCR was performed in triplicate using an iQ5 Optical System (Bio-Rad, Hercules, CA). Amplification was carried out as follows: a single denaturing step at 95°C for 10 min followed by 40 cycles of 95°C for 15 sec, 59°C for 30 sec, and 72°C for 30 sec, followed by a final extension step of 72°C for 3 min. Results were analyzed using the Relative Expression Software Tool (REST) [32, 33].

### 2.3. Type 1 Diabetes Assessment

Glucosuria was monitored weekly using Diastix reagent strips (Bayer, Elkhart, IN). Mice were considered diabetic after two consecutive positive tests, and the date of the first positive test was recorded as the time of onset of disease.

### 2.4. Flow Cytometry

Splenocytes from NOD.*β*_2_m^KO^.HHD and NOD.*β*_2_m^KO^.HHD.Ins2^het^ mice were analyzed by flow cytometry. Cells were stained with anti-B220, anti-CD11c, anti-CD4, and anti-CD8 (all from BD Biosciences, San Jose, CA). In some samples, cells were stained with anti-CD25 (BD Biosciences), fixed and permeabilized with fixation/permeabilization buffer (eBioscience, San Diego, CA), and stained with anti-Foxp3 (eBioscience).

### 2.5. Pancreas Histology

To assess insulitis in female NOD.*β*_2_m^KO^.HHD and NOD.*β*_2_m^KO^.HHD.Ins2^het^ mice at 4 and 8 weeks of age, pancreata were fixed in Bouin's solution, embedded in paraffin, and sectioned at nonoverlapping levels. Sections were stained with aldehyde fuchsine to readily visualize granulated beta cells and counterstained with hematoxylin and eosin for detection of leukocytes. Islets were scored as previously described [34]: 0, no insulitis; 1, local insulitis without infiltration of islet itself; 2, less than 25% infiltration; 3, 25–75% infiltration; or 4, greater than 75% infiltration. An insulitis index was calculated by adding the scores of all islets and dividing by four times the number of islets scored. A minimum of 20 islets per mouse were evaluated. Diabetic mice were assigned an insulitis index of 1.

### 2.6. Islet Isolation and Culture of Islet-Infiltrating T Cells

Islets were isolated from female NOD.*β*_2_m^KO^.HHD.Ins2^het^ mice at 8 weeks of age by collagenase P perfusion of the common bile duct as previously described [35]. Islets were handpicked using a micromanipulator and a dissecting microscope and up to 50 islets were transferred per well to 24-well plates in 500 *μ*L R-10 medium (RPMI 1640 (Invitrogen, Carlsbad, CA) containing 10% FBS, 1 mM sodium pyruvate, 28 *μ*M *β*-mercaptoethanol, 1x nonessential amino acids (Invitrogen)) with 50 U/mL recombinant human IL-2 (PeproTech, Rocky Hill, NJ). Cells were cultured for 7 days at 37°C in 5% CO_2_, at which point the majority of the cells are expected to be CD8 T cells [35].

### 2.7. IFN-*γ* ELISPOT Assay

Human HLA-A^*∗*^02:01-positive T2 cells [36], deficient for the transporter associated with antigen processing, were cultured at 26°C overnight prior to use. ELISPOT plates (Millipore MAHA S4510, Billerica, MA) were coated with anti-mouse IFN*γ* antibody (BD Biosciences) and blocked with 1% bovine serum albumin (Sigma-Aldrich, St. Louis, MO). T2 cells were plated at 2 × 10^4^ cells/well and pulsed with 10 *μ*M of the indicated peptides for 1 hour at 26°C. Cultured islet-infiltrating T cells from NOD.*β*_2_m^KO^.HHD.Ins2^het^ mice were added at 2 × 10^4^ cells/well in 50 *μ*L R-10. Cells were incubated for 40 hours at 37°C. Wells were then washed with 0.05% Tween 20/PBS and biotinylated anti-mouse IFN*γ* detection antibody (BD Biosciences) was added for 2 hours at 37°C. After washing, streptavidin-alkaline phosphatase (Zymed Laboratories, Carlsbad, CA) was added and incubated for 1 hour at 37°C. Wells were washed and spots were developed using 5-bromo-4-chloro-3-indolyl-phosphate/nitro-blue tetrazolium substrate (Sigma-Aldrich). Spots were counted using an automated ELISPOT reader system (Autoimmun Diagnostika, Strassberg, Germany). Responses are reported as a stimulation index, which is defined as spot number in response to the test peptide divided by spot number in response to an irrelevant HIV-derived HLA-A^∗^02:01-binding peptide (SLYNTVATL) [37]. The cutoff for positivity is a stimulation index greater than 2 and a test peptide spot number greater than 5 per 1 × 10^5^ T cells [38].

## 3. Results

### 3.1. Accelerated Diabetes Development in NOD.*β*_2_m^KO^.HHD.Ins2^het^ Mice

**Figure 1:**
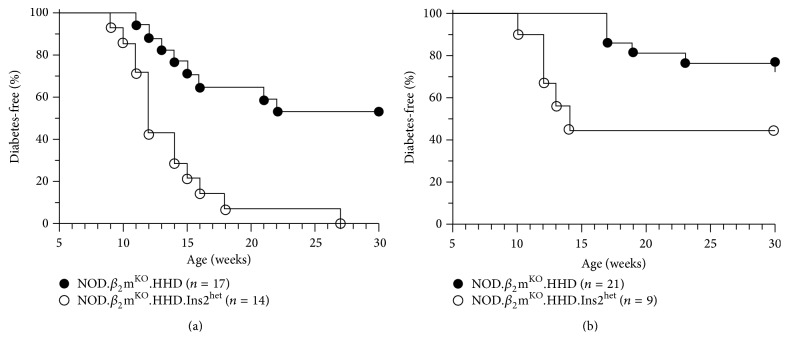
Diabetes development in NOD.*β*_2_m^KO^.HHD and NOD.*β*_2_m^KO^.HHD.Ins2^het^ mice. (a) Female and (b) male NOD.*β*_2_m^KO^.HHD (filled circles) and NOD.*β*_2_m^KO^.HHD.Ins2^het^ mice (open circles) were followed weekly for diabetes development. (a) *p* = 0.0002, Mantel-Cox. (b) *p* = 0.04, Mantel-Cox.

**Figure 2:**
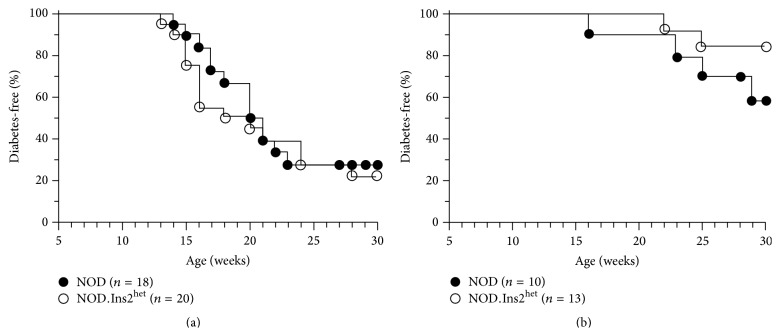
Diabetes development in NOD and NOD.Ins2^het^ mice. (a) Female and (b) male NOD (filled circles) and NOD.Ins2^het^ mice (open circles) were followed weekly for diabetes development. (a) *p* = 0.63 (not significant), Mantel-Cox. (b) *p* = 0.19 (not significant), Mantel-Cox.

A previous study had demonstrated that Ins2^het^ mice of mixed, but primarily C57BL/6, background experience a reduction in thymic insulin expression of approximately 40% [24]. To develop a mouse model of T1D having reduced thymic insulin quantity, and also expressing the human class I MHC molecule HLA-A^∗^02:01, we generated NOD.*β*_2_m^KO^.HHD.Ins2^het^ mice. Using quantitative RT-PCR, we similarly found a reduction in thymic insulin expression of 35% in female Ins2^het^ compared to Ins2^WT^ mice (*n* = 4 mice of each genotype). Diabetes development in NOD.*β*_2_m^KO^.HHD.Ins2^het^ and NOD.*β*_2_m^KO^.HHD mice of both genders was then compared. Both female (Figure 1(a)) and male NOD.*β*_2_m^KO^.HHD.Ins2^het^ mice (Figure 1(b)) demonstrated accelerated diabetes development compared to their Ins2^WT^ counterparts. Female NOD.*β*_2_m^KO^.HHD.Ins2^het^ mice developed diabetes as early as 9 weeks of age and all were diabetic by 27 weeks (Figure 1(a)). The first onset of diabetes in Ins2^WT^ female mice was at 11 weeks, and only 47% developed diabetes by 30 weeks. As also seen in standard NOD males [39, 40], diabetes development was slowed and overall incidence was reduced in NOD.*β*_2_m^KO^.HHD males (Figure 1(b)) compared to females. However, Ins2^het^ males exhibited an earlier onset of disease compared to Ins2^WT^ males (10 weeks versus 17 weeks), and a larger percentage (56% versus 24%) had developed diabetes by 30 weeks of age (Figure 1(b)). Thus, both genders of NOD.*β*_2_m^KO^.HHD.Ins2^het^ mice faithfully model the circumstance in humans where reduced thymic insulin expression is predisposing to T1D [16, 17]. Note that this is not what we observed in the case of NOD.Ins2^het^ mice, where both female (Figure 2(a)) and male Ins2^het^ mice (Figure 2(b)) exhibit a diabetes profile that is statistically indistinguishable from that of NOD mice.

### 3.2. Immune Cell Populations Are Not Grossly Altered in NOD.*β*_2_m^KO^.HHD.Ins2^het^ Mice

**Figure 3:**
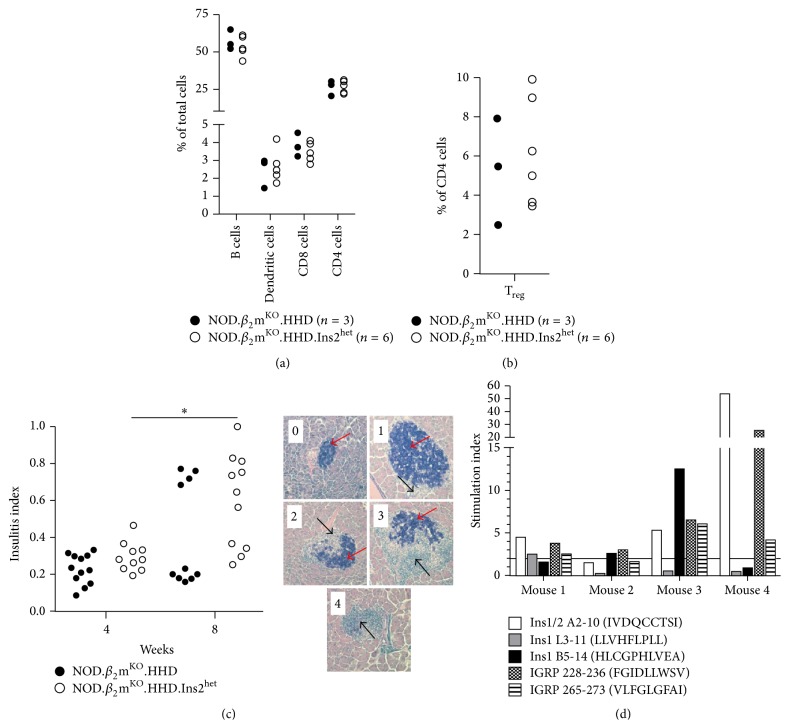
Splenocyte composition, insulitis, and autoreactive CD8 T cell specificities in NOD.*β*_2_m^KO^.HHD.Ins2^het^ mice. (a) and (b) Splenocytes from 8-week-old female NOD.*β*_2_m^KO^.HHD (filled circles) and NOD.*β*_2_m^KO^.HHD.Ins2^het^ mice (open circles) were analyzed by flow cytometry. Each symbol represents an individual mouse. (c) Female NOD.*β*_2_m^KO^.HHD (filled circles) and NOD.*β*_2_m^KO^.HHD.Ins2^het^ mice (open circles) were sacrificed at 4 and 8 weeks of age and insulitis indices were determined as described in Materials and Methods and plotted. Each symbol represents an individual mouse. ^*∗*^ *p* = 0.0037 (Mann-Whitney *U*). Representative islets from a single NOD.*β*_2_m^KO^.HHD.Ins2^het^ mouse are also shown. In these images, beta cells appear dark purple and are denoted by red arrows, while the more lightly stained infiltrating immune cells are marked by black arrows. The number on each image indicates the insulitis score of the islet shown. (d) Islet-infiltrating cells from 8-week-old female NOD.*β*_2_m^KO^.HHD.Ins2^het^ mice were tested for reactivity to the indicated HLA-A^*∗*^02:01-restricted insulin and IGRP epitopes by IFN-*γ* ELISPOT. Stimulation index was calculated by dividing the number of spots detected for a given peptide by the number of spots detected with an irrelevant HIV-derived HLA-A^*∗*^02:01-binding peptide. A stimulation index greater than 2 was considered a positive response.

To verify that the accelerated diabetes development observed in NOD.*β*_2_m^KO^.HHD.Ins2^het^ mice could not be attributed to a gross alteration in immune cell populations, we examined the splenocyte composition of 8-week-old female nondiabetic NOD.*β*_2_m^KO^.HHD and NOD.*β*_2_m^KO^.HHD.Ins2^het^ mice (Figure 3(a)). It was previously shown that NOD.*β*_2_m^KO^.HHD mice have a reduced CD8 T cell population and elevated B and CD4 T cells compared to standard NOD mice [31]. This was also true for NOD.*β*_2_m^KO^.HHD.Ins2^het^ mice, and no differences were observed in any of the cell types analyzed as a percentage of total cells. To investigate whether a reduction in regulatory T cells (T_reg_) might contribute to disease pathogenesis in the Ins2^het^ mice, NOD.*β*_2_m^KO^.HHD and NOD.*β*_2_m^KO^.HHD.Ins2^het^ splenocytes were monitored for expression of the characteristic T_reg_ cell phenotype, CD4^+^CD25^+^Foxp3^+^. No difference was observed in T_reg_ cells as a percentage of CD4 T cells (Figure 3(b)). These results indicate that the accelerated diabetes development seen in NOD.*β*_2_m^KO^.HHD.Ins2^het^ mice is the result of neither an altered immune cell composition nor reduced T_reg_ cells, at least at the level investigated here, that is, without regard to antigenic specificity.

### 3.3. NOD.*β*_2_m^KO^.HHD.Ins2^het^ Mice Exhibit Typical Signs of Islet Autoimmunity

In mixed background mice carrying zero, one, or two copies of the* Ins2* gene, pancreatic insulin content is indistinguishable [24]. Furthermore, Ins2^KO^ mice perform identically to their Ins2^WT^ counterparts in intraperitoneal glucose tolerance tests [41]. Thus, we hypothesized that the diabetes observed in NOD.*β*_2_m^KO^.HHD.Ins2^het^ mice was of an autoimmune nature, as is the case for the NOD.*β*_2_m^KO^.HHD parent strain [31], and not a deficiency in pancreatic insulin production due to the presence of only one functional copy of the* Ins2* gene. To verify this, histological sections of pancreata from female mice at 4 and 8 weeks of age were examined. All mice studied exhibited some degree of insulitis, which progressed significantly with age (Figure 3(c)), and islets showing a wide range of immune cell infiltration and beta cell destruction were observed (Figure 3(c)).

We previously identified several HLA-A^∗^02:01-restricted beta cell epitopes, derived from the autoantigens insulin and islet-specific glucose-6-phosphatase catalytic subunit-related protein (IGRP) that are recognized by islet-infiltrating T cells from NOD.*β*_2_m^KO^.HHD mice [31, 42]. To further confirm the autoimmune nature of the diabetes observed in NOD.*β*_2_m^KO^.HHD.Ins2^het^ mice, islets from 8-week-old females were cultured for 7 days and T cell reactivity to the previously identified beta cell epitopes was monitored by IFN*γ* ELISPOT. All mice harbored autoreactive T cells specific for at least two epitopes (Figure 3(d)), further confirming the autoimmune nature of their disease. A subset of these epitopes (Ins B5–14, IGRP 228–236, and IGRP 265–273) have previously been shown to be recognized by CD8 T cells in HLA-A^*∗*^02:01-positive T1D patients [43–46], supporting the clinical relevance of the model.

## 4. Discussion

Insulin is an important autoantigen recognized by T cells in both human T1D and the NOD mouse model of the disease [47]. Reduced thymic insulin expression is associated with susceptibility to T1D in patients [16, 17, 19, 20], suggesting that impaired negative selection of T cells specific for insulin is responsible for this predisposition. Here we have developed and characterized NOD.*β*_2_m^KO^.HHD.Ins2^het^ mice as a model of T1D that incorporates reduced thymic insulin. We find that, as in patients, disease is accelerated (Figure 1), and we suggest these mice as a new diabetes model that can be used to better understand this phenomenon. The NOD.*β*_2_m^KO^.HHD.Ins2^het^ mice present advantages over other disease models that have been described for this purpose. For example, thymic insulin expression is abolished in NOD.Ins2^KO^ and NOD.*β*_2_m^KO^.HHD.Ins2^KO^ mice, and both exhibit accelerated T1D [28–30] and increased insulin-specific islet-infiltrating CD8 T cells [28] when compared to their Ins2^WT^ counterparts. While these findings suggest the importance of thymic insulin expression, Ins2^KO^ models do not accurately represent patients, where thymic insulin expression is reduced, but not eliminated [19, 20]. As for NOD.Ins2^het^ mice, in our hands neither females nor males show accelerated disease (Figure 2). Two earlier studies of NOD.Ins2^het^ mice also showed no effect on disease in males [29, 30], and only one of the two showed acceleration in females [29]. In contrast, both male and female NOD.*β*_2_m^KO^.HHD.Ins2^het^ mice show enhanced disease (Figure 1). Indeed, the female and male incidence curves are nearly overlapping until 15 weeks of age (cf. Figures 1(a) and 1(b)). Thus, future mechanistic studies could realistically be performed using both genders. These studies should include the quantification of insulin-specific effector T cells and T_reg_ and analysis of their phenotype and function. The recently described ability to isolate insulin-specific CD4 T cells from NOD mouse strains using enrichment with peptide/MHC tetramer reagents will facilitate this work [48].

In NOD mice, establishment of immunological tolerance to insulin can lead to prevention of T1D [49–51] and remission of established disease [52]. Because of these findings, there is great interest in immunological interventions for human T1D that seek to manipulate the T cell response to insulin [53]. The NOD.*β*_2_m^KO^.HHD.Ins2^het^ mouse strain should be considered as an additional preclinical model to be used to evaluate such therapies, as it incorporates aspects of the human disease that are not represented in standard NOD mice, including reduced thymic insulin expression. In humans, VNTR alleles associated with diminished thymic insulin have been shown to alter the frequency and avidity of insulin-specific T cells [21], both of which could reasonably influence the outcome of therapies designed to manipulate the immune response to insulin. Given that human insulin-specific CD8 T cells have been shown to have cytotoxic activity against islets [54], an additional advantage of the NOD.*β*_2_m^KO^.HHD.Ins2^het^ mouse model is the expression of the T1D-predisposing human class I MHC allele HLA-A^*∗*^02:01 [4, 6, 8, 12], which we have shown as supporting the development of T cells specific for HLA-A^*∗*^02:01-restricted insulin epitopes in these mice (Figure 3(d)). In terms of insulin-specific CD4 T cells, the class II MHC allele expressed in the NOD.*β*_2_m^KO^.HHD.Ins2^het^ mice is I-A^g7^, which is structurally similar to the human T1D-predisposing HLA-DQ8 [55, 56]. Indeed, I-A^g7^ and HLA-DQ8 are capable of presenting similar peptides [57–59]. The NOD.*β*_2_m^KO^.HHD.Ins2^het^ mouse therefore has a variety of potential uses as a humanized model of T1D, including CD8 and CD4 T cell epitope identification, analysis of the relationship between thymic insulin expression and tolerance, and the evaluation of antigen-specific immunotherapies, particularly those targeting the immune response to insulin.

## 5. Conclusions

NOD.*β*_2_m^KO^.HHD.Ins2^het^ mice represent a model for T1D that incorporates the reduced, but not abolished, thymic insulin expression observed in patients. This model should find utility in investigations to probe the mechanisms underlying the association between reduced thymic insulin expression and T1D in humans. It will also be an important tool for T cell epitope discovery and for the preclinical evaluation of insulin-targeted immunotherapies for the disease.
